# Maternal intake of seafood and supplementary long chain n-3 poly-unsaturated fatty acids and preterm delivery

**DOI:** 10.1186/s12884-017-1225-8

**Published:** 2017-01-19

**Authors:** Anne Lise Brantsæter, Linda Englund-Ögge, Margareta Haugen, Bryndis Eva Birgisdottir, Helle Katrine Knutsen, Verena Sengpiel, Ronny Myhre, Jan Alexander, Roy M. Nilsen, Bo Jacobsson, Helle Margrete Meltzer

**Affiliations:** 1 Department of Environmental Exposure and Epidemiology, Domain of Infection Control and Environmental Health, Norwegian Institute of Public Health, P.O. Box 4404, Nydalen, NO-0403 Oslo, Norway; 2 Department of Obstetrics and Gynecology, Sahlgrenska University Hospital, Gothenburg, Sweden; 3 Unit for Nutrition Research, Landspitali University Hospital and University of Iceland, Reykjavik, Iceland; 4 Department of Genetics and Bioinformatics, Domain of Health Data and Digitalisation, Norwegian Institute of Public Health, Oslo, Norway; 5 Office of the Director-General, Norwegian Institute of Public Health, Oslo, Norway; 6 Department of Health and Social Sciences, Bergen University College, Bergen, Norway; 7 Department of Obstetrics and Gynecology, Sahlgrenska Academy, Gothenburg University, Gothenburg, Sweden

**Keywords:** Preterm delivery, Seafood consumption, Food frequency questionnaire, The Norwegian Mother and Child Cohort Study, MoBa

## Abstract

**Background:**

Preterm delivery increases the risk of neonatal morbidity and mortality. Studies suggest that maternal diet may affect the prevalence of preterm delivery. The aim of this study was to assess whether maternal intakes of seafood and marine long chain n-3 polyunsaturated fatty acids (LCn-3PUFA) from supplements were associated with preterm delivery.

**Methods:**

The study population included 67,007 women from the Norwegian Mother and Child Cohort Study. Maternal food and supplement intakes were assessed by a validated self-reported food frequency questionnaire in mid-pregnancy. Information about gestational duration was obtained from the Medical Birth Registry of Norway. We used Cox regression to estimate hazard ratios (HR) with 95% confidence intervals (CI) for associations between total seafood, lean fish, fatty fish, and LCn-3PUFA intakes and preterm delivery. Preterm was defined as any onset of delivery before gestational week 37, and as spontaneous or iatrogenic deliveries and as preterm delivery at early, moderate, and late preterm gestations.

**Results:**

Lean fish constituted 56%, fatty fish 34% and shellfish 10% of seafood intake. Any intake of seafood above no/rare intake (>5 g/d) was associated with lower prevalence of preterm delivery. Adjusted HRs were 0.76 (CI: 0.66, 0.88) for 1–2 servings/week (20–40 g/d), 0.72 (CI: 0.62, 0.83) for 2–3 servings/week (40–60 g/d), and 0.72 (CI: 0.61, 0.85) for ≥3 servings/week (>60 g/d), *p*-trend <0.001. The association was seen for lean fish (*p*-trend: 0.005) but not for fatty fish (*p*-trend: 0.411). The intake of supplementary LCn-3PUFA was associated only with lower prevalence of early preterm delivery (before 32 gestational weeks), while increasing intake of LCn-3PUFA from food was associated with lower prevalence of overall preterm delivery (*p*-trend: 0.002). Any seafood intake above no/rare was associated with lower prevalence of both spontaneous and iatrogenic preterm delivery, and with lower prevalence of late preterm delivery.

**Conclusions:**

Any intake of seafood above no/rare consumption was associated with lower prevalence of preterm delivery. The association was stronger for lean than for fatty fish. Intake of supplementary LCn-3PUFA was associated only with early preterm delivery. The findings corroborate the current advice to include fish and seafood as part of a balanced diet during pregnancy.

**Electronic supplementary material:**

The online version of this article (doi:10.1186/s12884-017-1225-8) contains supplementary material, which is available to authorized users.

## Background

Preterm delivery, which is defined as spontaneous or iatrogenic delivery before gestational week 37, is the major cause of perinatal mortality and morbidity and is an important risk factor of long-term physical and mental disabilities [1–4]. In Scandinavia and some other European countries, the rate is around 5–7% of all deliveries, while in the United States it is as high as 12% [5].

Preterm delivery accounts for a high financial burden on healthcare and is a considerable trauma for those involved [6–8]. Several factors have been shown to be associated with preterm delivery, including maternal demographic characteristics, reproductive history, infection, and biological and genetic markers [1, 9–11]. However, the aetiologies for preterm delivery are largely unknown and currently there is no effective treatment to reduce the rate of preterm delivery. Hence, it is important to identify potential modifiable factors in order to prevent the complications and cost associated with preterm delivery.

Seafood is a rich source of essential nutrients including protein, selenium, iodine, vitamin D, and the marine long chain n-3 polyunsaturated fatty acids (LCn-3PUFA) which have important structural and physiological roles in the body, including neurological, immune, and cardiovascular systems [12–14].

In prospective observational studies, high levels of maternal fish consumption during pregnancy have been associated with longer gestation [15–17] and lower prevalence of preterm delivery [16, 18], but the results are not found in all studies [19–21]. The beneficial effects of fish consumption have primarily been attributed to the LCn-3PUFA eicosapentaenoic acid (EPA) and docosahexaenoic acid (DHA). Randomized controlled trials have shown lower risk of preterm delivery, and particularly early preterm delivery (<34 weeks) in women supplemented with EPA and DHA during pregnancy [22–25].

The current dietary advice to pregnant women in Norway and other countries is to include lean and fatty fish as part of a balanced diet and to limit or avoid consumption of contaminated species [26–28]. In Norway there is a long tradition not only for eating seafood, but also for use of cod liver oil. More studies of maternal seafood and LCn-3PUFA intake in relation to preterm delivery are needed to disentangle, if possible, the role of different types of fish and supplementary LCn-3PUFA [29, 30]. In the Norwegian Mother and Child Cohort Study, women reported in detail their intakes of food and dietary supplements during pregnancy, making it possible to quantify their total seafood consumption and subcategories of lean and fatty fish, as well as LCn-3PUFA contributed by use of dietary supplements and LCn-3PUFA contributed by food (i.e., fish) [31–34].

The aim of the present study was to examine associations of maternal seafood and LCn-3PUFA supplement intakes with the risk of preterm delivery. We hypothesized that higher intake of seafood is associated with lower risk of preterm delivery and that associations vary by seafood categories. We investigated the associations with all preterm deliveries and with the outcome stratified as spontaneous and iatrogenic preterm delivery and as early, moderate and late preterm delivery.

## Methods

### Population and study design

The Norwegian Mother and Child Cohort Study (MoBa) is a prospective population-based pregnancy cohort study conducted by the Norwegian Institute of Public Health [35]. Participants were recruited from across Norway from 1999 through 2008, and 40.6% of the invited women participated. The cohort now includes 114,500 children, 95,200 mothers and 75,200 fathers. Women were recruited to the study by postal invitation before the routine free ultrasound examination around gestational week 18. The women were asked to provide blood and urine samples at baseline and to answer questionnaires at regular intervals during pregnancy and after birth. Follow-up is conducted by questionnaires at regular intervals and by linkage to national health registries [35, 36].

The data included in this study were from two questionnaires answered around gestational weeks 17 (questionnaire 1) [37] and 22 (questionnaire 2) [38]. Questionnaire 1 was a general questionnaire covering lifestyle, background, illness and health-related factors. Questionnaire 2 was a semi-quantitative food frequency questionnaire (FFQ), in which women reported their dietary habits from the start of the pregnancy. The response rates for the questionnaires during pregnancy were 95% for questionnaire 1 and 92% for questionnaire 2 [39]. Pregnancy and birth records from the Medical Birth Registry of Norway (MBRN) are linked to the MoBa database [36].

**Fig. 1:**
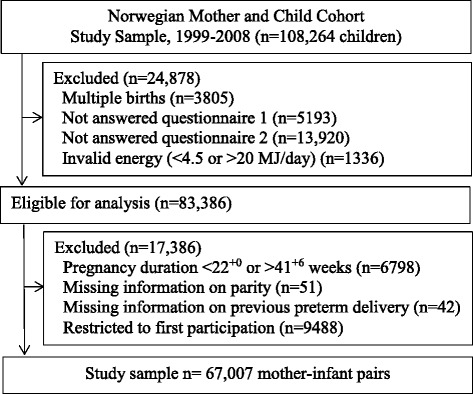
Flow chart showing selection of the study participants from the Norwegian Mother and Child Cohort Study

The current study is based on version 5 of the quality-assured data files released for research in 2010 (*n* = 108,264). To be included in the study, participants had to have delivered a live, singleton baby and to have answered both the first general questionnaire and the FFQ. They also had to have a valid energy intake between 4.5 and 20 MJ/day, resulting in *n* = 83,386 eligible for analysis. We excluded women with a duration of pregnancy less than 22^+0^ or more than 41^+6^ weeks (+days) (*n* = 6798), those with missing information about parity (*n* = 51) and those with missing information about previous preterm delivery (*n* = 42). To avoid the use of multiple dependent observations in our analyses, women who participated in the cohort more than once (*n* = 9488) were only included with their first participation, resulting in a final study sample of 67,007 mother-infant pairs (Fig. 1).

### Dietary assessment

The MoBa FFQ was completed around the 22nd week of gestation, and the dietary data were collected from February 2002 and onwards [32]. The MoBa FFQ is a semi-quantitative questionnaire designed to capture dietary habits during the first 4 to 5 months of pregnancy [38]. The FFQ included questions about intake of 255 food items with special emphasis on various seafood items. There were 10 questions about cold cuts and spreads made of fish or shellfish, 16 questions about fish or shellfish eaten for dinner, and four questions about cod liver oil, cod liver oil capsules or fish oil capsules.

Nutrient calculations were performed with the use of FoodCalc [40] and the Norwegian food composition table [41]. In the FFQ, the women were asked to record the use of dietary supplements. We have developed a database for nutrient content in more than 1000 dietary supplements reported by MoBa participants. For calculating intake of LCn-3PUFA from supplements we used name and brand name combined with reported frequency and amount. The FFQ has been thoroughly validated with regard to nutrients, foods and dietary supplements [31, 33, 42].

### Definition of fish and seafood variables and LCn-3PUFA intake

The daily intakes (g/day) of fish items were grouped as lean fish or fatty fish and included items eaten as bread spread, in salad, as dinner or as part of a mixed dish such as fish fingers or fish au gratin. In composite fish dishes, only the fish part of the dish was included in the calculated fish intake. Lean fish species included cod, saithe, haddock, pollock, halibut, plaice, flounder, tuna, perch, pike, Atlantic cat fish and fish roe (0.3–6.0% fat). Fatty fish species included mackerel, herring, salmon and trout (10–24% fat). We also calculated the intakes of shellfish (shrimp, crab and mussels (0.8–2.5% fat) and fish liver. Total seafood was the combined intake of lean fish, fatty fish, shellfish, and fish liver.

When evaluating the association between seafood consumption and preterm delivery, seafood intake was treated both as a continuous variable (g/d) and divided into the following categories: 0–5 g/d, >5- ≤ 20 g/d, >20- ≤ 40 g/ d, >40- ≤ 60 g/d, and ≥60 g/d. The same categories were used in two previous studies that examined seafood intake in relation to infant size at birth [34, 43]. Assuming a serving size of 140 g, these categories correspond to; never/rare intake, <1 serving/week, 1to <2 servings/week, 2 to <3 servings/week and 3 or more servings/week. When seafood was eaten as bread spread, a serving size was estimated to be 20–25 g. Lean and fatty fish variables were examined as continuous variables and divided into the five categories described above. In adjusted analyses, these variables were mutually adjusted (i.e., both entered in the same models). Lean and fatty fish intakes correlated with r_s_ = 0.22 (*p* < 0.001), while correlations for lean and fatty fish with shellfish and fish liver were weaker.

LCn-3PUFA in the current study was defined as the sum of eicosapentaenoic acid (EPA) and docosahexaenoic acid (DHA). The amount (mg/d) of LCn-3PUFA contributed by dietary supplements (EPA and DHA in cod liver oil, fish oil, cod liver oil capsules and fish oil capsules) and the amount of LCn-3PUFA contributed by fish (EPA and DHA) was estimated from the FFQ. The variables for LCn-3PUFA (i.e., amount contributed from food and amount contributed from supplements) were examined as continuous and as ranked variables. LCn-3PUFA from food and the total sum of LCn-3PUFA from food and supplements were divided into quintiles. In all models with seafood intake as the exposure, we adjusted for the amount of LCn-3PUFA from supplements divided into three categories with non-users as one group and consumers ranked into two groups divided by median.

### Preterm delivery

We defined preterm delivery as delivery before gestational week 37^+0^ and used this as the primary outcome. Gestational age in days was obtained from the MBRN and determined by second-trimester ultrasound in 98.2% of pregnancies and based on the last menstrual period in the remaining cases [36]. Preterm delivery was categorised based on delivery initiation, i.e., spontaneous preterm delivery (preterm labour or preterm prelabour rupture of the membranes) or iatrogenic preterm delivery (induced or primary caesarean delivery on maternal or foetal indications). Preterm delivery was also categorised into late (34^+0^ to 36^+6^ weeks), moderately (32^+0^ to 33^+6^ weeks) and early preterm (22^+0^ to 31^+6^ weeks).

### Other variables

We included a range of potential covariates and examined their association with seafood intake and preterm delivery. Information about maternal age at delivery and previous preterm delivery was obtained from the MBRN. We treated maternal age as a continuous variable. History of previous preterm delivery was analysed as a dichotomous variable (yes/no). Body mass index (BMI) was calculated from prepregnancy weight and height that women had reported in questionnaire 1. We only included women with pre-pregnancy weight in the range 35–180 kg and height above 1.40 m. BMI was divided into four categories (<18.5, 18.5–24.9, 25–29.9, ≥ 30 kg/m^2^) and a missing category (*n* = 1723). Height was divided into quartiles. Information about parity came from questionnaire 1 and from MBRN and was divided into two categories (nulliparous or parous). Information about marital status, smoking in pregnancy, maternal education and household income was obtained from questionnaire 1. Marital status was divided into living alone or cohabiting and smoking into yes (occasional or daily smoker) or no. Maternal education was divided into four categories: ≤12 years (high school or less), 13–16 years (3–4 years of college/university), 17 + years (4 years or more of college/university), or other/missing (*n* = 1418). Household income was expressed as a combination of the participant’s and her partner’s annual income and divided into three categories: both partners <300,000 NOK, one partner ≥300,000 NOK, both partners ≥300,000 NOK, or missing information (*n* = 1936). Information about alcohol intake and whether or not the pregnancy was planned was divided into yes or no. We used total energy intake (kJ) as a continuous variable.

### Statistical methods

We used one-way analysis of variance for continuous variables, chi-square test for categorical variables and Mann-Whitney *U* test for nominal data to test differences between groups. The main exposure variables were total seafood, including the subcategories lean and fatty fish, and LCn-3PUFA from food and supplements. We used Cox regression to estimate hazard ratios (HR) for preterm delivery with 95% confidence intervals (CI). Preterm delivery was the defined event and gestational days the underlying time variable with day 153 as the entry time (22 completed weeks of gestation). Follow-up ended at the date of preterm delivery or at 259 days of gestation (36 completed weeks of gestation), whichever came first. In the separate analysis of spontaneous and iatrogenic preterm delivery, the other category was censored but kept in the analysis. *P* for trend was obtained by incorporating the categorical variables as linear terms in the models. Variables included in the adjusted models were: maternal age, education, history of previous preterm delivery, height, BMI, marital status, parity, smoking, household income and total energy intake.

Repeating the analyses using logistic regression resulted in odds ratios comparable to the hazard ratios obtained by Cox regression. Hazard ratios and odds ratios represent different association measures, but both approximate relative risks when the outcome is rare [44].

We examined the associations between seafood and preterm delivery separately in women with prepregnant BMI < 25 and those with prepregnant BMI ≥ 25 kg/m^2^, and separately for nulliparous and parous pregnancies. We also conducted other sensitivity analyses, including complete case analysis.

We used visual inspection of the log-log plot to verify that the proportional-hazard assumption was essentially fulfilled All analyses were performed using PASW Statistics software version 19 for Windows (SPSS Inc., IBM Company, Chicago Ill., USA). All *P* values were two sided and values below 0.05 were considered statistically significant.

## Results

Consumption of any seafood was reported by 97.7% of all women in this study, 5.7% reported no lean fish, 13% reported no fatty fish, 10.7% reported no shellfish and 91% reported no consumption of fish liver. The median intakes of total seafood, lean fish and fatty fish were 33.4, 18.5 and 8.3 g/day, respectively. Lean and fatty fish constituted on average 56 and 34% of the total seafood intake (Table 1). The median total seafood intake corresponds to 1–2 servings per week. The median amount of LCn-3PUFA from total seafood was 0.25 g/d (mean 0.37 g/d), with median 0.039 g/d from lean fish (mean 0.044 g/d)) and 0.188 g/d from fatty fish (mean 0.298 g/d). On average, fatty fish contributed 75% and lean fish 11% to the LCn-3PUFA intake from food (Table 1). Use of LCn-3PUFA supplements was reported by 67% of the women. The supplement users were divided into two groups based on the median intake of supplemental LCn-3PUFA (<0.30 g/d and ≥0.30 g/d). In the low group, LCn-3PUFA intakes ranged from 0.01 g/d-0.29 g/d (median 0.16 g/d) and in the high group from 0.30 g/d-8.8 g/d (median 0.80 g/d).

**Table 1:** Calculated intake seafood and LCn-3PUFA from food and % contribution from subcategories in *n =* 67,007 women in the Norwegian Mother and Child Cohort Study 2002–2008

	Median, g/d	Mean, g/d	% of total seafood	LCn-3PUFA, median g/d	LCn-3PUFA, mean g/d	% of LCn-3PUFA
Total seafood	33.4	36.4		0.248	0.365	92
Lean fish	18.5	20.3	56	0.039	0.044	11
Fatty fish	8.3	12.2	34	0.188	0.298	75
Shellfish	2.4	3.6	10	0	0.009	2.2
Fish liver	0	0.3	0.7	0	0.014	3.7
Eggs^a^	7.8	11.4		0.014	0.019	4.8
Other food (poultry)^a^	18.8	22.4		0.010	0.012	3.0

^a^Eggs and other food contain LCn-3PUFA (eicosapentaenoic and docosahexaenoic acid) coming from marine feed ingredients

Maternal characteristics differed across increasing categories of seafood intake and between LCn-3PUFA non-supplement and supplement users (Table 2). Maternal age and energy intake increased with increasing seafood intake, while BMI decreased. Women in the lowest consumption categories included more nulliparous women and more women with low education and income than to those in the higher consumption categories. Increasing seafood intake was associated with not only increasing intakes of fish and shellfish, but also by increasing intakes of LCn-3PUFA from food and from supplements. LCn-3PUFA supplement users had lower BMI, included fewer first time mothers and fewer smokers, and included more women with high education and income than non-supplement users (Table 2).

**Table 2:** Seafood consumption and LCn-3PUFA supplement use by maternal characteristics. *N* = 67,007 women in the Norwegian Mother and Child Cohort Study 2002–2008

	Seafood consumption (g/day)						LCn-3PUFA^b^ supplement use		
	0–5 (4.4%)	>5–20 (18.4%)	>20–40 (40.4%)	>40–60 (24.5%)	>60 (12.3%)	*p-value* ^*a*^	No (32.9%)	Yes (67.1%)	*p-value* ^a^
*Continuous Variables (mean and standard deviation)*									
Maternal age at delivery (years)	28 (5)	29 (5)	30 (4)	31 (4)	31 (5)	*<0.001*	30 (5)	30 (4)	*<0.001*
Maternal height (m)^b^	1.67 (0.06)	1.68 (0.06)	1.68 (0.06)	1.68 (0.06)	1.68 (0.06)	*<0.001*	1.68 (0.06)	1.68 (0.06)	*<0.001*
Prepregnancy body mass index (kg/m^2^)^c^	24.4 (4.6)	24.2 (4.4)	24.0 (4.2)	23.9 (4.2)	23.9 (4.3)	*<0.001*	24.6 (4.6)	23.7 (4.0)	*<0.001*
Total energy intake (MJ)	9.3 (2.8)	9.3 (2.5)	9.6 (2.5)	9.9 (2.5)	10.7 (2.9)	*<0.001*	9.7 (2.7)	9.7 (2.5)	*0.216*
Lean fish (g/day)	0.5 (1.1)	8.1 (4.5)	18.0 (7.3)	28.3 (10.5)	37.0 (17.3)	*<0.001*	20.4 (14.5)	20.2 (13.2)	*0.196*
Fatty fish (g/day)	0.5 (1.1)	4.0 (3.5)	8.6 (6.0)	15.4 (9.9)	34.4 (24.1)	*<0.001*	11.4 (14.1)	12.6 (14.1)	*<0.001*
Shellfish (g/day)	0.3 (0.8)	1.9 (2.4)	3.3 (4.5)	4.5 (5.2)	6.8 (10.9)	*<0.001*	3.6 (6.0)	3.7 (5.2)	*0.405*
LCn-3PUFA^b^ from food (g/day)	0.09 (0.05)	0.21 (0.10)	0.36 (0.18)	0.57 (0.29)	1.18 (0.77)	*<0.001*	0.45 (0.45)	0.48 (0.44)	*<0.001*
LCn-3PUFA^b^ from supplements (g/day)	0.29 (0.54)	0.32 (0.51)	0.37 (0.54)	0.40 (0.58)	0.44 (0.62)	*<0.001*	0	0.56 (0.60)	*<0.001*
*Discrete characteristics (%)* ^b^									
Nulliparous mother (%)	38.4	41.7	48.1	52.7	52.5	*<0.001*	39.5	57.9	*<0.001*
Previous preterm delivery (%)	2.9	3.4	3.4	3.9	4.1	*0.002*	4.6	3.1	*<0.001*
Single mother (%)	5.8	3.8	3.3	3.9	4.9	*<0.001*	4.7	3.5	*<0.001*
Maternal smoking (%)^c^	14.2	8.8	7.3	7.5	8.9	*<0.001*	12.9	5.8	*<0.001*
Maternal education^c^									
≤ 12 years	48.3	34.7	28.3	28.5	34.4	*<0.001*	43.2	25.2	*<0.001*
13–16 years	33.2	40.2	43.3	42.8	38.2		37.4	43.6	
> 16 years	15.5	22.6	26.5	26.9	25.0		17.0	29.2	
Household income (annual)^c^									
Both partners < NOK 300,000	37.5	30.0	27.2	27.5	30.5	*<0.001*	34.3	24.2	*<0.001*
One partner ≥ NOK 300,000	42.3	42.3	41.9	42.3	42.8		41.9	40.6	
Both partners ≥ NOK 300,000	20.2	27.7	30.8	30.1	26.7		20.0	32.2	
Planned pregnancy^c^	74.6	80.6	81.4	80.7	77.8	*<0.001*	76.6	82.2	*<0.001*

^a^ *P* value for difference: One-way analysis of variance for continuous variables, chi-square test for categorical variables

^b^LCn-3PUFA: marine long chain n-3 polyunsaturated fatty acids

^c^Missing information prepregnant body mass index: 2.6%, maternal education: 2.1%, household income: 2.9%, planned pregnancy:1.1%

The overall proportion of preterm delivery in the study population was 5.4% (*n* = 3630), comprising 3.1% (*n* = 2051) spontaneous and 2.2% (*n* = 1419) iatrogenic preterm deliveries, while information about delivery initiation was missing for 88 preterm cases (0.1%). Of all deliveries, 2659 (4.0%) were late preterm deliveries, 491 (0.7%) were moderately preterm and 480 (0.7%) were early preterm deliveries (Table 3). In crude analyses using continuous seafood intake variables, women with preterm deliveries had lower intakes of total seafood, lean fish and fatty fish than women with term deliveries. This was also found for LCn-3PUFA from food, while there was no difference in the amount of LCn-3PUFA from supplements between the groups. The differences in seafood intakes were seen particularly for the subcategories spontaneous and iatrogenic preterm delivery and for late preterm delivery (Table 3).

**Table 3:** Maternal intake (g/day) of seafood and marine long chain n-3 polyunsaturated fatty acids (LCn-3PUFA) from food and dietary supplements in 67,007 women with and without preterm delivery (PTD) in the Norwegian Mother and Child Cohort Study (MoBa) 2002–2008

	N (%)	Total seafood	Lean fish	Fatty fish	Food LCn-3PUFA	Suppl. LCn-3PUFA
		Median (P5, P95)	Median (P5, P95)	Median (P5, P95)	Median (P5, P95)	Median (P5, P95)
Overall preterm delivery						
*No*	63,377 (94.6)	33.5 (6.2, 75.9)	18.5 (0, 45.2)	8.3 (0, 38.3)	0.35 (0.10, 1.27)	0.16 (0, 1.60)
*Yes*	3630 (5.4)	32.1 (3.5, 78.6)	17.7 (0, 44.8)	7.6 (0, 40.3)	0.34 (0.08, 1.32)	0.16 (0, 1.60)
*Crude p-value* ^a^		*<0.001*	*<0.001*	*<0.001*	*<0.001*	*0.936*
*Adjusted p-value* ^b^		*0.004*	*0.008*	*0.349*	*0.171*	*0.879*
*Subcategories by delivery initiation* ^c^						
Spontaneous preterm deliveries						
No	63,377 (94.6)	33.5 (6.2, 75.9)	18.5 (0, 45.2)	8.3 (0, 38.3)	0.35 (0.10, 1.27)	0.16 (0, 1.60)
Yes	2051 (3.1)	31.7 (3.0, 78.4)	17.9 (0, 45.1)	7.6 (0, 39.3)	0.33 (0.08, 1.32)	0.16 (0, 1.60)
*Crude p-value*		*<0.001*	*0.011*	*0.001*	*<0.001*	*0.680*
Iatrogenic preterm delivery						
No	63,377 (94.6)	33.5 (6.2, 75.9)	18.5 (0, 45.2)	8.3 (0, 38.3)	0.35 (0.10, 1.27)	0.16 (0, 1.60)
Yes	1491 (2.2)	33.0 (4.1, 78.6)	17.5 (0, 45.3)	7.6 (0, 42.6)	0.35 (0.09, 1.33)	0.15 (0, 1.60)
*Crude p-value*		*0.031*	*0.008*	*0.045*	*0.123*	*0.294*
*Subcategories by week of delivery*						
Late PTD (35 to <37 w)						
No	63,377 (94.6)	33.5 (6.2, 75.9)	18.5 (0, 45.2)	8.3 (0, 38.3)	0.35 (0.10, 1.27)	0.16 (0, 1.60)
Yes	2659 (4.0)	31.8 (3.5, 78.3)	17.7 (0, 44.1)	7.6 (0, 39.6)	0.34 (0.09, 1.32)	0.16 (0, 1.60)
*Crude p-value*		*<0.001*	*<0.001*	*0.002*	*0.001*	*0.720*
Moderately PTD *(32 to <34w)*						
No	63,377 (94.6)	33.5 (6.2, 75.9)	18.5 (0, 45.2)	8.3 (0, 38.3)	0.35 (0.10, 1.27)	0.16 (0, 1.60)
Yes	491 (0.7)	33.6 (3.0, 81.0)	17.5 (0, 45.5)	8.1 (0, 44.1)	0.35 (0.08, 1.44)	0.15 (0, 1.60)
*Crude p-value*		*0.530*	*0.155*	*0.378*	*0.712*	*0.386*
Early PTD *(22 to <32 w)*						
No	63,377 (94.6)	33.5 (6.2, 75.9)	18.5 (0, 45.2)	8.3 (0, 38.3)	0.35 (0.10, 1.27)	0.16 (0, 1.60)
Yes	480 (0.7)	32.0 (3.3, 78.0)	18.1 (0, 45.8)	7.2 (0, 38.6)	0.33 (0.09, 1.23)	0.14 (0, 1.60)
*Crude p-value*		*0.102*	*0.467*	*0.018*	*0.016*	*0.050*

P5: 5th percentile, P95: 95th percentile

^a^Crude *P-values* from non-parametric Mann-Whitney *U* test

^b^Adjusted *P*-values from Cox regression with continuous seafood variables adjusted for the other seafood and LCn-3PUFA variables when relevant, maternal age, pre-pregnancy BMI, height, parity, energy intake, maternal education, smoking, marital status, household income and previous preterm delivery

^c^Missing data on delivery initiation for 88 preterm deliveries

**Fig. 2:**
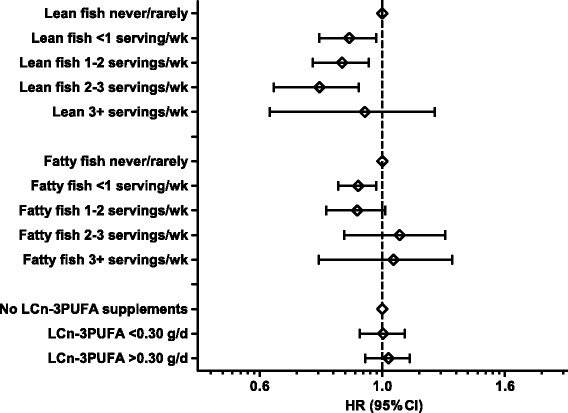
Associations (hazard ratio (HR) and 95% confidence intervals (CI)) between intakes of lean fish, fatty fish and marine long chain n-3 polyunsaturated fatty acids (LCn-3PUFA) from supplements and preterm delivery. Intakes are mutually adjusted and adjusted for maternal age, pre-pregnancy BMI, height, parity, energy intake, maternal education, smoking, marital status, household income and previous preterm delivery. *N* = 67,007 women in the Norwegian Mother and Child Cohort Study (MoBa) 2002–2008

Examining seafood intake by categories with the never/rarely as the reference category, showed lower prevalence of preterm delivery for all other intake categories (Table 4). For total seafood intake, the lowest risk estimates were observed in the two highest intake categories with adjusted HR: 0.72 (95%CI: 0.62, 0.83) for 2- ≤ 3 servings per week and HR: 0.72 (95%CI: 0.61, 0.85) for ≥3 servings per week, *p*-trend <0.001 (Table 3). When lean and fatty fish were examined as separate variables, significantly lower risk for preterm delivery was observed for lean fish intake in all intake categories except the highest category (*p*-trend: 0.005). For fatty fish, lower risk was seen only for the intake category corresponding to 1- ≤ 2 servings per week (*p*-trend 0.411). LCn-3PUFA from supplements was not associated with overall preterm delivery (Fig. 2 and Table 4).

**Table 4:** Associations between total seafood intake, lean and fatty fish intake and marine long chain n-3 polyunsaturated fatty acids (LCn-3PUFA) from supplements and preterm delivery (PTD). *N* = 67,007 women in the Norwegian Mother and Child Cohort Study (MoBa) 2002–2008

	All	PTD	Unadjusted	Adjusted
	n	n (%)	HR^a^ (95% CI)	HR^ab^ (95% CI)
Total seafood				
≤ 5 g/d (never/rarely)	2966	220 (7.4)	1	1
> 5–20 g/d (<1 serving/week)	12,299	713 (5.8)	0.77 (0.66, 0.90)	0.80 (0.69, 0.93)
> 20–40 g/d (1–2 servings/week)	27,045	1440 (5.3)	0.71 (0.62, 0.82)	0.76 (0.66, 0.88)
> 40–60 g/d (2–3 servings/week)	16,432	824 (5.0)	0.67 (0.57, 0.77)	0.72 (0.62, 0.83)
> 60 g/d (≥3 servings/week)	8265	433 (5.2)	0.70 (0.59, 0.82)	0.72 (0.61, 0.85)
*P* for trend^c^			*<0.001*	*<0.001*
Lean fish				
≤ 5 g/d (never/rarely)	7453	492 (6.6)	1	1
> 5–20 g/d (<1 serving/week)	29,159	1578 (5.4)	0.82 (0.74, 0.90)	0.88 (0.79, 0.98)
> 20–40 g/d (1–2 servings/week)	24,834	1277 (5.1)	0.77 (0.70, 0.86)	0.86 (0.77, 0.95)
> 40–60 g/d (2–3 servings/week)	4799	236 (4.9)	0.74 (0.63, 0.86)	0.78 (0.67, 0.92)
> 60 g/d (≥3 servings/week)	762	47 (6.2)	0.93 (0.69, 1.26)	0.91 (0.67, 1.23)
*P* for trend^c^			*<0.001*	*0.005*
Fatty fish				
≤ 5 g/d (never/rarely)	22,035	1305 (5.9)	1	1
> 5–20 g/d (<1 serving/week)	33,297	1693 (5.1)	0.85 (0.80, 0.92)	0.91 (0.85, 0.98)
> 20–40 g/d (1–2 servings/week)	8654	448 (5.2)	0.87 (0.78, 0.97)	0.91 (0.81, 1.02)
> 40–60 g/d (2–3 servings/week)	1898	116 (6.1)	1.03 (0.85, 1.25)	1.06 (0.87, 1.28)
> 60 g/d (≥3 servings/week)	1123	68 (6.1)	1.03 (0.80, 1.31)	1.02 (0.80, 1.31)
*P* for trend^c^			*0.088*	*0.411*
LCn-3PUFA from supplements				
No supplement	22,018	1093 (5.4)	1	1
< 0.30 g/d (median 0.16 g/d)	22,493	1207 (5.4)	0.99 (0.92, 1.07)	1.00 (0.92, 1.09)
≥ 0.30 g/d (median 0.80 g/d)	22,496	1230 (5.5)	1.01 (0.93, 1.09)	1.02 (0.94, 1.11)
*P* for trend^c^			*0.818*	*0.567*

^a^HR: Hazard Ratio (Cox regression)

^b^Adjusted for the other seafood categories and LCn-3PUFA from supplements, maternal age, pre-pregnancy BMI, height, parity, energy intake, maternal education, smoking, marital status, household income and previous preterm delivery

^c^P for linear trend obtained by incorporating variable as linear term

We further examined whether the associations between LCn-3PUFA from supplements and preterm delivery differed between groups of women with low and high seafood intake. Low seafood intake was defined as no seafood intake and intakes below the 5th percentile. Total seafood intake was also ranked into tertiles, quintiles and deciles, and the association between LCn-3PUFA from supplements and preterm delivery examined in each strata separately. No significant association between LCn-3PUFA from supplements and preterm delivery was observed at any level of seafood intake (results not shown). Furthermore, we examined LCn-3PUFA from supplements as a continuous variable and ranked into quintiles and deciles. No significant associations with preterm delivery were indicated (results not shown).

We repeated the analysis using the calculated LCn-3PUFA intake from food as the exposure and the lowest quintile as reference category. Lower risk estimates were seen in all the other quintiles (Additional file 1: Table S1), with the lowest HR observed in quintile 4 (HR 0.83, CI: 0.75, 0.92), *p*-trend 0.002. However, when we summed the intake of LCn-3PUFA from food and supplements, the association with preterm delivery was significant only in the third quintile (Additional file 1: Table S1). When LCn-3PUFA from food was included in a model with seafood as the exposure, the hazard ratios for seafood and preterm delivery remained significant, but with wider confidence intervals (i.e., for 2–3 servings/week HR: 0.77 (95%CI: 0.64, 0.92) and for ≥3 servings/week HR: 0.75 (95%CI: 0.62, 0.92).

We further analysed associations between seafood intake and subcategories of preterm delivery. Any intake of seafood beyond no/rare intake was associated with lower prevalence of both spontaneous and iatrogenic preterm deliveries (Table 5), with HRs similar to those seen for any preterm (20–30% risk reduction). In these analyses, lean fish had a stronger influence on the association for iatrogenic than for spontaneous preterm delivery (*p*-trend: 0.012 for iatrogenic vs 0.219 for spontaneous), while fatty fish and LCn-3PUFA from supplements did not show a significant trend with either spontaneous or iatrogenic preterm delivery.

**Table 5:** Associations between seafood intake and marine long chain n-3 polyunsaturated fatty acids (LCn-3PUFA) from supplements and the subcategories spontaneous and iatrogenic preterm delivery. *N* = 66,919^a^ women in the Norwegian Mother and Child Cohort Study (MoBa) 2002–2008

	All	*Spontaneous*	Adjusted	*Iatrogenic*	Adjusted
	n	PTD n (%)	HR^ab^ (95% CI)	PTD n (%)	HR^bc^ (95% CI)
		2051 (3.1)		1491 (2.2)	
Total seafood					
≤ 5 g/d (never/rarely)	2961	129 (4.4)	1	86 (2.9)	1
> 5–20 g/d (<1 serving/week)	12,275	405 (3.3)	0.79 (0.64, 0.96)	284 (2.3)	0.81 (0.63, 1.03)
> 20–40 g/d (1–2 servings/week)	27,010	802 (3.0)	0.73 (0.61, 0.89)	603 (2.2)	0.79 (0.63, 0.99)
> 40–60 g/d (2–3 servings/week)	16,416	469 (2.9)	0.71 (0.58, 0.86)	339 (2.1)	0.74 (0.58, 0.94)
> 60 g/d (≥3 servings/week)	8257	246 (3.0)	0.73 (0.58, 0.90)	179 (2.2)	0.71 (0.55, 0.92)
*P* for trend^d^			*0.009*		*0.013*
Lean fish					
≤ 5 g/d (never/rarely)	7437	278 (3.7)	1	198 (2.7)	1
> 5–20 g/d (<1 serving/week)	29,118	884 (3.0)	0.88 (0.77, 1.02)	653 (2.2)	0.89 (0.76, 1.05)
> 20–40 g/d (1–2 servings/week)	24,806	723 (2.9)	0.88 (0.76, 1.02)	526 (2.1)	0.84 (0.71, 0.99)
> 40–60 g/d (2–3 servings/week)	4797	139 (2.9)	0.85 (0.69, 1.04)	95 (2.0)	0.74 (0.57, 0.95)
> 60 g/d (≥3 servings/week)	761	27 (3.5)	0.99 (0.67, 1.47)	19 (2.5)	0.81 (0.50, 1.31)
*P* for trend^c^			*0.219*		*0.012*
Fatty fish					
≤ 5 g/d (never/rarely)	22,003	746 (3.4)	1	527 (2.4)	1
> 5–20 g/d (<1 serving/week)	33,257	949 (2.9)	0.89 (0.81, 0.99)	704 (2.1)	0.94 (0.84, 1.06)
> 20–40 g/d (1–2 servings/week)	8640	256 (3.0)	0.91 (0.79, 1.06)	178 (2.1)	0.89 (0.75, 1.06)
> 40–60 g/d (2–3 servings/week)	1897	59 (3.1)	0.94 (0.71, 1.23)	56 (3.0)	1.26 (0.95, 1.66)
> 60 g/d (≥3 servings/week)	1122	41 (3.7)	1.10 (0.80, 1.51)	26 (2.3)	0.94 (0.63, 1.39)
*P* for trend^c^			*0.411*		*0.804*
LCn-3PUFA from supplements					
No supplement	21,999	671 (3.1)	1	503 (2.3)	1
< 0.30 g/d (median 0.16 g/d)	22,456	671 (3.0)	0.97 (0.87, 1.09)	499 (2.2)	1.03 (0.90, 1.17)
≥ 0.30 g/d (median 0.80 g/d)	22,464	709 (3.2)	1.03 (0.92, 1.15)	489 (2.2)	1.01 (0.89, 1.15)
*P* for trend^d^			*0.626*		*0.872*

^a^Missing data on delivery initiation for 88 preterm deliveries

^b^HR: Hazard Ratio (Cox regression)

^c^Adjusted for the other seafood categories and LCn-3PUFA from supplements, maternal age, pre-pregnancy BMI, height, parity, total energy intake, maternal education, smoking, marital status, household income and previous preterm delivery

^d^ *P* for linear trend obtained by incorporating variable as linear term

When preterm delivery was examined separately according to late, moderate or early onset, any increase in seafood beyond no/rare intake was associated with lower risk in the late preterm group, with HRs resulting in 20–30% risk reduction (*p*-trend <0.001) (Table 6). Although not statistically significant, the risk estimates for moderately and early preterm delivery were comparable to those for late preterm delivery. In these analyses LCn-3PUFA from supplements was not associated with late or moderately preterm delivery, while a non-significant trend was indicated for early preterm delivery, HR 0.84 (CI: 0.67, 1.05) and HR 0.81 (CI: 0.65, 1.01) for the two supplement intake groups versus no LCn-3PUFA from supplements (*p*-trend 0.072) (Table 6).

**Table 6:** Associations between total seafood intake and the subcategories late preterm delivery (35 to <37 w), moderately preterm delivery (32 to <34 w) and early preterm delivery (22 to <32 w). *N* = 67,007 mothers in the Norwegian Mother and Child Cohort Study (MoBa) 2002–2008

	All	PTD	Unadjusted	Adjusted
		n (%)	HR^a^ (95% CI)	HR^b^ (95% CI)
*Late PTD (35 to <37 w)*		2659 (4.0)		
Total seafood intake				
≤ 5 g/d (never/rarely)	2903	157 (5.4)	1	1
> 5–20 g/d (<1 serving/week)	12,111	525 (4.3)	0.80 (0.67, 0.95)	0.82 (0.68, 0.98)
> 20–40 g/d (1–2 servings/week)	26,684	1079 (4.0)	0.74 (0.63, 0.88)	0.78 (0.66, 0.92)
> 40–60 g/d (2–3 servings/week)	16,194	586 (3.6)	0.66 (0.55, 0.79)	0.69 (0.58, 0.83)
> 60 g/d (≥3 servings/week)	8144	312 (3.8)	0.70 (0.58, 0.85)	0.71 (0.58, 0.86)
*P* for trend^c^			*<0.001*	*<0.001*
LCn-3PUFA from supplements				
No supplement	21,676	851 (3.9)	1	1
< 0.30 g/d (median 0.16 g/d)	22,203	917 (4.1)	1.05 (0.96, 1.16)	1.07 (0.97, 1.18)
≥ 0.30 g/d (median 0.80 g/d)	22,157	891 (4.0)	1.03 (0.93, 1.13)	1.04 (0.94, 1.15)
*P* for trend^c^			*0.609*	*0.450*
*Moderately PTD (32 to <34w)*		491 (0.7)		
Total seafood intake				
≤ 5 g/d (never/rarely)	2778	32 (1.2)	1	1
> 5–20 g/d (<1 serving/week)	11,680	94 (0.8)	0.70 (0.47, 1.04)	0.75 (0.50, 1.12)
> 20–40 g/d (1–2 servings/week)	25,784	179 (0.7)	0.60 (0.41, 0.88)	0.68 (0.47, 0.99)
> 40–60 g/d (2–3 servings/week)	15,730	122 (0.8)	0.67 (0.46, 0.99)	0.77 (0.52, 1.15)
> 60 g/d (≥3 servings/week)	7896	64 (0.8)	0.70 (0.46, 1.07)	0.78 (0.51, 1.20)
*P* for trend^c^			*0.432*	*0.792*
LCn-3PUFA from supplements				
No supplement	20,987	169 (0.8)	1	1
< 0.30 g/d (median 0.16 g/d)	21,424	138 (0.6)	0.83 (0.66, 1.05)	0.84 (0.66, 1.06)
≥ 0.30 g/d (median 0.80 g/d)	21,457	191 (0.9)	1.15 (0.94, 1.42)	1.19 (0.95, 1.48)
*P* for trend^c^			*0.158*	*0.093*
*Early PTD (22 to <32 w)*		480 (0.7)		
Total seafood intake				
≤ 5 g/d (never/rarely)	2777	31 (1.1)	1	1
> 5–20 g/d (<1 serving/week)	11,680	94 (0.8)	0.72 (0.48, 1.08)	0.77 (0.51, 1.16)
> 20–40 g/d (1–2 servings/week)	25,787	182 (0.7)	0.63 (0.43, 0.92)	0.71 (0.48, 1.04)
> 40–60 g/d (2–3 servings/week)	15,724	116 (0.7)	0.66 (0.44, 0.98)	0.75 (0.50, 1.12)
> 60 g/d (≥3 servings/week)	7899	57 (0.7)	0.65 (0.42, 0.99)	0.68 (0.43, 1.06)
*P* for trend^c^			*0.116*	*0.196*
LCn-3PUFA from supplements				
No supplement	21,005	180 (0.9)	1	1
< 0.30 g/d (median 0.16 g/d)	21,438	152 (0.7)	0.83 (0.67, 1.03)	0.84 (0.67, 1.05)
≥ 0.30 g/d (median 0.80 g/d)	21,414	148 (0.7)	0.81 (0.65, 1.00)	0.81 (0.65, 1.01)
*P* for trend^c^			*0.049*	*0.072*

^a^HR: Hazard Ratio (Cox regression)

^b^Adjusted for maternal age, pre-pregnancy BMI, height, parity, energy intake, maternal education, smoking, marital status, household income, previous preterm delivery and LCn-3PUFA from supplements

^c^ *P* for linear trend obtained by incorporating variable as linear term

Statistical significance of the adjusted associations was similar whether the seafood and LCn-3PUFA variables were modelled as continuous or categorical variables.

Obesity is a risk factor of preterm delivery, and we examined the associations between seafood intake and preterm delivery separately in women with pre-pregnant BMI <25 (*n* = 45,119) and those with BMI ≥ 25 kg/m^2^ (*n* = 20,165). The prevalence of preterm delivery was 5.0% in the group with BMI <25 and 6.3% in the groups with BMI ≥25 kg/m^2^. Lower risk of preterm delivery with seafood intake above 1 serving a week (20-30% risk reduction) was seen in both strata, although slightly stronger HRs were observed in women with BMI <25 than in those with BMI ≥25 kg/m^2^ (Additional file 2: Table S2).

In our study sample, preterm delivery was more prevalent in nulliparous (6.3%) than in parous women (4.3%), and we also examined the associations between seafood intake and preterm delivery separately in nulliparous (*n* = 34,731) and parous women (*n* = 32,276). Lower HRs for preterm delivery were seen in both strata, but the effect size was smaller and the confidence intervals wider in parous than in nulliparous women (Additional file 2: Table S2). LCn-3PUFA from dietary supplements was not associated with preterm delivery in any of these analyses (Additional file 2: Table S2 and Additional file 3: Table S3).

Finally we tested whether the associations between seafood intake and preterm delivery were consistent in subcategories of maternal age, education and smoking. The risk estimates were comparable in all sub-strata, but in small groups e.g., smokers, the confidence intervals were wider (results not shown).

Excluding women who had a registered diagnosis of diabetes in the MBRN (*n* = 1007) or adjusting for diabetes as an independent variable did not change the associations between seafood and LCn-3PUFA and preterm delivery (results not shown).

## Discussion

The main finding in the present study was the significant association between maternal total seafood intake and lower likelihood of preterm delivery. The association was mainly explained by intake of lean fish. The results for the calculated intake of LCn-3PUFA from food reflected the results for seafood intake, while LCn-3PUFA from supplements was not associated with the outcome, with the exception of a borderline significant trend for early preterm delivery. Lower prevalence of preterm delivery with increasing seafood intake was observed for both the subcategories spontaneous and iatrogenic preterm delivery. Significantly lower HR was observed for moderately increased intakes relative to no/low intake, while we observed no additional risk reduction for the highest intake categories.

A beneficial relationship between seafood consumption and preterm delivery has been reported in several studies [15–18, 45]. In a previous study in nulliparous women in MoBa focusing on the Mediterranean diet, we found that eating fish at least twice weekly during pregnancy was associated with lower risk of preterm delivery [46]. We defined Mediterranean diet according to five a-priori defined criteria, and associations with preterm delivery were examined for adherence to each criterion and for all criteria combined. Only the criterion ‘eating fish at least twice weekly’ was significantly associated with the outcome (adjusted OR: 0.84; 95% CI: 0.74, 0.95) [46]. A more recent study from MoBa examining maternal dietary patterns showed a reduced risk of preterm delivery associated with high adherence to a traditional dietary pattern characterised by i.e., potatoes, fish dishes and lean fish [47]. The present study complements the previous studies by including more detailed quantification of seafood intakes, by separating between lean and fatty fish intakes, and by examining LCn-3PUFA from food and supplements as independent exposure variables.

Associations between fish consumption and LCn-3PUFA with increased length of gestation observed in previous studies have been explained by the anti-inflammatory properties of these fatty acids modulating the inflammatory pathways leading to cervical ripening and initiation of labour and delivery [48–51]. Klebanoff et al. (2011) studied fish consumption, erythrocyte fatty acids, and preterm delivery in a high-risk population of women with a prior preterm delivery. The women participated in a randomized controlled trial of n-3 supplementation [52]. Women were randomized to receive either an LCn-3PUFA supplement or placebo starting in mid-gestation (weeks 16–21). The results showed that women who reported the lowest fish consumption at gestational weeks 16–21 had higher risk of recurrent preterm birth than those who ate fish more frequently. The lowest occurrence of preterm birth was seen among women who ate fish approximately 2–3 times/week, while more frequent fish consumption was not associated with further risk reduction [52]. Interestingly, the lowest occurrence of preterm birth was observed among women in the second quartile of erythrocyte LCn-3PUFA concentrations, and no benefit of LCn-3PUFA supplementation was found, regardless of baseline fish consumption and erythrocyte n-3 concentration [52]. A multicentre randomized control trial in seven European countries comprising women with previous pregnancy complications found that fish oil supplementation (2.7 g LCn-3PUFA daily) delayed the onset of delivery for women with low (mean 16 g/d) and medium fish intake (mean 23 g/day), but not for women with high fish intake (mean 36 g/day) at baseline [53], suggesting that the effect of fish oil supplementation on timing of delivery depends on a woman’s fish intake. In the current study, however, no association between LCn-3PUFA from supplements and overall preterm delivery was evident at any level of seafood intake. However, our finding of borderline significantly lower risk of early preterm delivery (<32 weeks) in women using LCn-3PUFA is in line with results from experimental studies in the US and Australia which found that supplementation with 0.6–0.8 g/d DHA reduced the prevalence of early preterm births in low risk pregnancies [22, 23]. Contrary to our findings, a prospective study in pregnant women in Massachusetts found no associations between intake of fish or LCn-3PUFA from food and preterm delivery [20]. Furthermore, another recent study from the US reported an increased risk of preterm in women with high intakes of lean fish [21].

In the present study, LCn-3PUFA from fish paralleled the results obtained for seafood, while LCn-3PUFA from supplements was not associated with overall preterm delivery. The association between seafood intake and preterm delivery remained significant also when adjusted for LCn-3PUFA from food, indicating that the observed associations between seafood consumption and lower prevalence of preterm delivery cannot be explained only by LCn-3PUFA. Alternative explanations could be other components in fish (e.g., proteins, iodine, and selenium) or other aspects of fish consumption (e.g., foods typically eaten with fish or foods displaced by fish) might modulate the inflammatory pathways leading to delivery. The effects of fatty fish, lean fish and LCn-3PUFA intake on inflammation have been studied in some intervention studies [54, 55]. In one study, participants were randomly allocated to receive dietary advice plus either 300 g of fatty fish (salmon) or 300 g of lean fish (cod) per week for 6 months, or only dietary advice. Interestingly, the effect estimates did not differ between the lean and fatty fish groups, and a significantly lower concentration of the systemic inflammation marker C-reactive protein was found in both fish groups compared to the control group [54]. Another study investigated the effects of weight loss and seafood consumption in three intervention groups (salmon, cod, or LCn-3PUFA supplementation) and controls on inflammation parameters during energy restriction. The largest decrease in inflammation parameters was observed for salmon consumption, and no decrease was seen for LCn-3PUFA supplementation [55]. It could also be speculated that fish consumption may influence the gut microbiota which is crucial for optimal functioning of the digestive and immune systems [56]. Two experimental studies explored whether fish consumption influenced gut microbiota composition and local markers of gut inflammation without convincing results [54, 57]. However, in one of these studies a significant effect on the systemic inflammatory markers was observed for both lean and fatty fish [54].

An issue of particular concern when it comes to seafood consumption in women of childbearing age, is the concomitant exposure to environmental pollutants, e.g., methylmercury, polychlorinated biphenyls and perfluorinated alkylated substances, which may counteract the beneficial effects of fish consumption [28, 58–61]. However, a systematic review of environmental contaminant exposures and preterm birth found no consistent evidence for chemical exposure and increased risk of preterm delivery [62]. Likewise, studies in MoBa examining associations between maternal exposure to environmental pollutants primarily contributed by seafood and pregnancy outcomes showed no increased risk of preterm delivery [63, 64]. The result of the present study corroborates the current advice to pregnant women to include fish and seafood as part of a balanced diet and restricting the intake of species and items with known high concentrations of environmental pollutants [26, 65].

The strengths of this study include the prospective design and detailed information about maternal diet, demography, socioeconomic factors, and pregnancy outcomes. Thanks to the large sample size we were able to examine the associations between seafood intake and preterm delivery in subcategories. Participants in MoBa were recruited from urban and rural, coastal and inland regions and represented different socioeconomic groups. Dietary intake was assessed using an FFQ that was specifically developed and validated for use in this cohort [31, 32]. The FFQ included questions with special emphasis on various seafood items, and the validation study showed that the reported intakes of lean and fatty fish and LCn-3PUFA supplements were reflected by blood and urine samples [33]. The food frequency method challenges respondents with rather complex cognitive tasks and are more suitable for ranking participants according to high and low intakes than for precise intake calculations. Therefore, differential misclassification of seafood and n-3 supplement intakes cannot be excluded, but is as likely to occur in women with term delivery as in women with preterm delivery.

The somewhat low response rate in MoBa is a concern and participants are not representative of all pregnant women in Norway. The potential bias due to self-selection has been evaluated by comparison of eight exposure-outcome associations reported both in MoBa and in the MBRN. Significant differences in prevalence estimates were found for most variables, e.g., MoBa participants included less smokers than the general pregnant population. In spite of this, there were no statistically relative differences in association measures, e.g., prenatal smoking and low birthweight [66]. It is a strength that the women who do participate remain in the study, illustrated by a response rate of more than 90% for the three extensive questionnaires answered during pregnancy [39]. However, the study is observational and we cannot rule out the possibility that residual or unmeasured confounding may still exist.

## Conclusions

This study showed that maternal seafood consumption was associated with reduced risk of preterm delivery. The association was mainly explained by intake of lean fish. The association was seen for subcategories spontaneous, iatrogenic and late preterm delivery. Intake of LCn-3PUFA from seafood was associated with preterm delivery, while intake of LCn-3PUFA from supplements was associated only with early preterm delivery. The association between supplementary LCn-3PUFA and preterm delivery did not differ between women with low or high seafood intake. Furthermore, the association between seafood intake and reduced risk of preterm delivery remained significant when adjusted for LCn-3PUFA from food, indicating that the observed associations cannot be explained only by LCn-3PUFA and that other properties related to fish and seafood consumption may also be of importance. The findings corroborate the current advice to include fish and seafood as part of a balanced diet during pregnancy. Preterm delivery is a serious public health problem resulting in high societal costs, and no effective preventive strategies for the general population exist. Dietary changes have low cost and low risk compared with medical interventions and even moderate changes may be of public health importance.

## Additional files

Additional file 1: Table S1. Associations between estimated intake of polyunsaturated fatty acids (LCn-3PUFA) from food and total LCn3-PUFA (food and supplements) and preterm delivery. (DOCX 30 kb)
Additional file 2: Table S2. Associations between total seafood intake and marine long chain polyunsaturated fatty acids (LCn-3PUFA) from supplements and preterm delivery in women stratified according to pre-pregnant BMI <25 (*n* = 45,119) or pre-pregnant BMI ≥ 25 kg/m2 (*n* = 20,165) in the Norwegian Mother and Child Cohort Study (MoBa) 2002–2008. (DOC 47 kb)
Additional file 3: Table S3. Associations between total seafood intake and marine long chain polyunsaturated fatty acids (LCn-3PUFA) from supplements and preterm delivery in women stratified according to nulliparous (*n* = 34,731) and parous women (*n* = 32,276) in the Norwegian Mother and Child Cohort Study (MoBa) 2002–2008. (DOC 43 kb)

## Abbreviations

BMI: Body Mass Index
CI: Confidence Interval
DHA: Docosahexaenoic acid
EPA: Eicosapentaenoic
FFQ: Food Frequency Questionnaire
HR: Hazard Ratio
LCn-3PUFA: Long Chain omega-3 Poly-Unsaturated Fatty Acids
MBRN: Medical Birth Registry of Norway
MoBa: The Norwegian Mother and Child Cohort Study
