# Erratum to: Maternal intake of seafood and supplementary long chain n-3 poly-unsaturated fatty acids and preterm delivery

**DOI:** 10.1186/s12884-017-1243-6

**Published:** 2017-02-10

**Authors:** Anne Lise Brantsæter, Linda Englund-Ögge, Margareta Haugen, Bryndis Eva Birgisdottir, Helle Katrine Knutsen, Verena Sengpiel, Ronny Myhre, Jan Alexander, Roy M. Nilsen, Bo Jacobsson, Helle Margrete Meltzer

**Affiliations:** 1 Department of Environmental Exposure and Epidemiology, Domain of Infection Control and Environmental Health, Norwegian Institute of Public Health, P.O. Box 4404Nydalen, NO-0403 Oslo, Norway; 2 Department of Obstetrics and Gynecology, Sahlgrenska University Hospital, Gothenburg, Sweden; 3 Unit for Nutrition Research, Landspitali University Hospital and University of Iceland, Reykjavik, Iceland; 4 Department of Genetics and Bioinformatics, Domain of Health Data and Digitalisation, Norwegian Institute of Public Health, Oslo, Norway; 5 Office of the Director-General, Norwegian Institute of Public Health, Oslo, Norway; 6 Department of Health and Social Sciences, Bergen University College, Bergen, Norway; 7 Department of Obstetrics and Gynecology, Sahlgrenska Academy, Gothenburg University, Gothenburg, Sweden

## Erratum

In the original publication of this article [1], the categories for lean and fatty fish in Fig. 2 should have been listed as servings per week and not per day. Please see updated figure below.

**Fig. 2:**
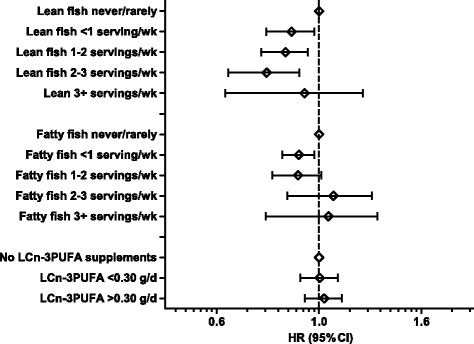
Associations (hazard ratio (HR) and 95% confidence intervals (CI)) between intakes of lean fish, fatty fish and marine long chain n-3 polyunsaturated fatty acids (LCn-3PUFA) from supplements and preterm delivery. Intakes are mutually adjusted and adjusted for maternal age, pre-pregnancy BMI, height, parity, energy intake, maternal education, smoking, marital status, household income and previous preterm delivery. *N* = 67,007 women in the Norwegian Mother and Child Cohort Study (MoBa) 2002–2008
