# Supplementary material for: Maternal intake of seafood and supplementary long chain n-3 poly-unsaturated fatty acids and preterm delivery
Source: BMC Pregnancy Childbirth. 2017 Jan 19;17:41. doi: 10.1186/s12884-017-1225-8 (PMC5248483; doi:10.1186/s12884-017-1225-8)
Supplement: Additional file 1: Table S1. — Associations between estimated intake of polyunsaturated fatty acids (LCn-3PUFA) from food and total LCn3-PUFA (food and supplements) and preterm delivery. (DOCX 30 kb) [file 12884_2017_1225_MOESM1_ESM.docx]

## Table S1 Associations between estimated intake of polyunsaturated fatty acids (LCn-3PUFA) from food and total LCn3-PUFA (food and supplements) and preterm delivery. N=67,007 mothers in the Norwegian Mother and Child Cohort Study (MoBa) 2002-2008

|  | All  n | PTD  n (%) | Unadjusted  HR^a^ (95% CI) | Adjusted  HR^b^ (95% CI) |
| --- | --- | --- | --- | --- |
| LCn-3PUFA from food |  |  |  |  |
| Quintile 1 (<0.20 g/d) | 13,396 | 844 (6.3) | 1 | 1 |
| Quintile 2 (0.20 – 0.29 g/d) | 13,399 | 716 (5.3) | 0.84 (0.76, 0.93) | 0.87 (0.79, 0.97) |
| Quintile 3 (0.30 – 0.41 g/d) | 13,406 | 680 (5.1) | 0.80 (0.72, 0.88) | 0.85 (0.77, 0.94) |
| Quintile 4 (0.42 – 0.64 g/d) | 13,404 | 675 (5.0) | 0.79 (0.72, 0.88) | 0.83 (0.75, 0.92) |
| Quintile 5 (>0.64 g/d) | 13,402 | 715 (5.3) | 0.84 (0.76, 0.93) | 0.85 (0.77, 0.95) |
| *P* for trend ^c^ |  |  | *<0.001* | *0.002* |
|  |  |  |  |  |
| LCn-3PUFA from food and supplements |  |  |  |  |
| Quintile 1 (<0.30 g/d) | 13,401 | 779 (5.8) | 1 | 1 |
| Quintile 2 (0.30 – 0.47 g/d) | 13,404 | 739 (5.5) | 0.95 (0.86, 1.05) | 0.96 (0.86, 1.06) |
| Quintile 3 (0.48 – 0.75 g/d) | 13,396 | 673 (5.0) | 0.86 (0.78, 0.95) | 0.87 (0.79, 0.97) |
| Quintile 4 (0.76 – 1.30 g/d) | 13,404 | 736 (5.5) | 0.94 (0.85, 1.04) | 0.94 (0.85, 1.04) |
| Quintile 5 (>1.30 g/d) | 13,402 | 703 (5.2) | 0.90 (0.81, 1.00) | 0.91 (0.82, 1.01) |
| *P* for trend ^c^ |  |  | *0.060* | *0.085* |

^a^ HR: Hazard Ratio (Cox regression). ^b^Adjusted for maternal age, pre-pregnancy BMI, height, parity, energy intake, maternal education, smoking, marital status, household income and previous preterm delivery. The model for LCn-3PUFA from food was also adjusted for LCn-3PUFA from supplements.

^c^ *P* for linear trend obtained by incorporating variable as linear term.
