# Supplementary material for: Maternal intake of seafood and supplementary long chain n-3 poly-unsaturated fatty acids and preterm delivery
Source: BMC Pregnancy Childbirth. 2017 Jan 19;17:41. doi: 10.1186/s12884-017-1225-8 (PMC5248483; doi:10.1186/s12884-017-1225-8)
Supplement: Additional file 2: Table S2. — Associations between total seafood intake and marine long chain polyunsaturated fatty acids (LCn-3PUFA) from supplements and preterm delivery in women stratified according to pre-pregnant BMI <25 (n = 45,119) or pre-pregnant BMI ≥ 25 kg/m2 (n = 20,165) in the Norwegian Mother and Child Cohort Study (MoBa) 2002–2008. (DOC 47 kb) [file 12884_2017_1225_MOESM2_ESM.doc]

## Table S2. Associations between total seafood intake and marine long chain polyunsaturated fatty acids (LCn-3PUFA) from supplements and preterm delivery in women stratified according to pre-pregnant BMI <25 (n=45,119) or pre-pregnant BMI ≥ 25 kg/m2 (n= 20,165) in the Norwegian Mother and Child Cohort Study (MoBa) 2002-2008.

|  | *BMI<25kg/m^2^*  n | PTD  n (%) | Adjusted  HR^ab^ (95% CI) | *BMI≥25kg/m^2^*  n | PTD  n (%) | Adjusted  HR^ab^ (95% CI) |
| --- | --- | --- | --- | --- | --- | --- |
|  |  | 2269 (5.0) |  |  | 1264 (6.3) |  |
| Total seafood |  |  |  |  |  |  |
| ≤ 5 g/d (never/rarely) | 1851 | 130 (7.0) | 1 | 1013 | 82 (8.1) | 1 |
| > 5 – 20 g/d (<1 serving/week) | 8045 | 442 (5.5) | 0.79 (0.65, 0.96) | 3950 | 256 (6.5) | 0.81 (0.63, 1.04) |
| > 20 – 40 g/d (1–2 servings/week) | 18,320 | 907 (5.0) | 0.74 (0.61, 0.89) | 8096 | 489 (6.0) | 0.77 (0.61, 0.98) |
| > 40 – 60 g/d (2–3 servings/week) | 11,253 | 513 (4.6) | 0.68 (0.56, 0.83) | 4728 | 290 (6.1) | 0.79 (0.62, 1.01) |
| > 60 g/d (≥3 servings/week) | 5650 | 277 (4.9) | 0.70 (0.57, 0.87) | 2378 | 147 (6.3) | 0.75 (0.56, 0.99) |
| *P* for trend ^c^ |  |  | *0.001* |  |  | *0.121* |
|  |  |  |  |  |  |  |
| LCn-3PUFA from supplements |  |  |  |  |  |  |
| No supplement | 13,296 | 660 (5.0) | 1 | 8001 | 490 (6.1) | 1 |
| < 0.30 g/d (<median) | 15,550 | 773 (5.0) | 0.99 (0.89, 1.11) | 6444 | 408 (6.3) | 1.02 (0.89, 1.16) |
| ≥ 0.30 g/d (≥median) | 16,273 | 836 (5.1) | 1.03 (0.92, 1.14) | 5720 | 366 (6.4) | 1.00 (0.87, 1.15) |
| *P* for trend ^c^ |  |  | *0.515* |  |  | *0.985* |

^a^ HR: Hazard Ratio (Cox regression). ^b^Adjusted for maternal age, pre-pregnancy BMI, height, parity, energy intake, maternal education, smoking, marital status, household income and previous preterm delivery. ^c^ *P* for linear trend obtained by incorporating variable as linear term.

Information about BMI was missing for n=1723 (2.6%) mothers.
