# Supplementary material for: Maternal intake of seafood and supplementary long chain n-3 poly-unsaturated fatty acids and preterm delivery
Source: BMC Pregnancy Childbirth. 2017 Jan 19;17:41. doi: 10.1186/s12884-017-1225-8 (PMC5248483; doi:10.1186/s12884-017-1225-8)
Supplement: Additional file 3: Table S3. — Associations between total seafood intake and marine long chain polyunsaturated fatty acids (LCn-3PUFA) from supplements and preterm delivery in women stratified according to nulliparous (n = 34,731) and parous women (n = 32,276) in the Norwegian Mother and Child Cohort Study (MoBa) 2002–2008. (DOC 43 kb) [file 12884_2017_1225_MOESM3_ESM.doc]

## Table S3. Associations between total seafood intake and marine long chain polyunsaturated fatty acids (LCn-3PUFA) from supplements and preterm delivery in women stratified according to nulliparous (n= 34,731) and parous women (n= 32,276) in the Norwegian Mother and Child Cohort Study (MoBa) 2002-2008.

|  | *Nulliparous* | PTD  n (%) | Adjusted  HR^ab^ (95% CI) | *Parous* | PTD  n (%) | Adjusted  HR^ab^ (95% CI) |
| --- | --- | --- | --- | --- | --- | --- |
|  | 34,731 | 2174 (6.3) |  | 32,276 | 1456 (4.5) |  |
| Total seafood |  |  |  |  |  |  |
| ≤ 5 g/d (never/rarely) | 1826 | 154 (8.4) | 1 | 1140 | 66 (5.8) | 1 |
| > 5 – 20 g/d (<1 serving/week) | 7176 | 463 (6.5) | 0.77 (0.64, 0.93) | 5123 | 250 (4.9) | 0.86 (0.65, 1.13) |
| > 20 – 40 g/d (1–2 servings/week) | 14,023 | 866 (6.2) | 0.74 (0.62, 0.88) | 13,022 | 574 (4.4) | 0.80 (0.61, 1.03) |
| > 40 – 60 g/d (2–3 servings/week) | 7780 | 449 (5.8) | 0.70 (0.58, 0.84) | 8652 | 375 (4.3) | 0.76 (0.58, 0.99) |
| > 60 g/d (≥3 servings/week) | 3926 | 242 (6.2) | 0.72 (0.59, 0.89) | 4339 | 191 (4.4) | 0.72 (0.56, 0.96) |
| *P* for trend ^c^ |  |  | *0.004* |  |  | *0.012* |
|  |  |  |  |  |  |  |
| LCn-3PUFA from supplements |  |  |  |  |  |  |
| No supplement | 8699 | 591 (6.8) | 1 | 13,319 | 602 (4.5) | 1 |
| < 0.30 g/d (<median) | 13,120 | 787 (6.0) | 0.94 (0.84, 1.05) | 9373 | 420 (4.5) | 1.10 (0.96, 1.25) |
| ≥ 0.30 g/d (≥median) | 12,912 | 796 (6.2) | 0.96 (0.86, 1.07) | 9584 | 434 (4.5) | 1.11 (0.97, 1.26) |
| *P* for trend ^c^ |  |  | *0.545* |  |  | *0.104* |

^a^ HR: Hazard Ratio (Cox regression). ^b^Adjusted for maternal age, pre-pregnancy BMI, height, parity, energy intake, maternal education, smoking, marital status, household income and previous preterm delivery. ^c^ *P* for linear trend obtained by incorporating variable as linear term.
